# Impact of COVID-19 on the incidence of visceral leishmaniasis in children and adolescents in the Northeast region, 2007-2022: time series study

**DOI:** 10.1590/S2237-96222025v34e20240382.en

**Published:** 2025-06-13

**Authors:** Pedro Henrique Ferreira dos Santos, Bruna Rykelly Ramos dos Santos, Karol Fireman de Farias

**Affiliations:** 1 Universidade Federal de Alagoas, Arapiraca, AL, Brazil

**Keywords:** Visceral Leishmaniasis, Child Health, Epidemiology, Neglected Diseases, Public Health, Leishmaniasis visceral, Salud infantil, Epidemiología, Enfermedades desatendidas, Salud pública

## Abstract

**Objective:**

To assess the impact of COVID-19 on the incidence of visceral leishmaniasis in children and adolescents living in the Northeast region of Brazil.

**Methods:**

Time series study with data from the Notifiable Diseases Information System, from the study of population estimates by municipality, age and sex from 2000 to 2021, and from the Brazilian Institute of Geography and Statistics. An analysis of the temporal trends of the incidence rate was performed using the Joinpoint software, calculating the average annual percentage variation (AAPV) and its 95% confidence intervals (95%CI). The percentage variation in incidence rates from 2020 to 2022 was also calculated to check whether they were below or above what was expected for the year and territory.

**Results:**

From 2007 to 2022, 13,890 new cases of visceral leishmaniasis were reported in children and adolescents in Northeast Brazil. Changes in trends were identified with the addition of the years of the COVID-19 pandemic, from 2020 to 2022, in the Northeast: AAPV (2007-2019) 0.44; 95%CI -2.23; 3.18; AAPV (2007-2022) -7.95; 95%CI -13.63; -1.88), Pernambuco: AAPV (2007-2019) 6.77; 95%CI 2.04; 11.71; AAPV (2007-2022) -1.40; 95%CI -8.75; 6.53 and Alagoas: AAPV (2007-2019) 11.51; 95%CI 4.90; 18.53; AAPV (2007-2022) -1.33; 95%CI -14.89; 14.40).

**Conclusion:**

The Northeast and its states where changes in temporal trends were observed after the COVID-19 pandemic are priorities for the intensification of actions to combat and control the disease.

## Resumen

**Objetivo:**

Evaluar el impacto de la covid-19 en la incidencia de leishmaniasis visceral en niños y adolescentes residentes en la región Nordeste de Brasil.

**Métodos:**

Estudio de series temporales con datos del Sistema de Información de Enfermedades de Declaración Obligatoria, del estudio de estimaciones de población por municipio, edad y sexo de 2000 a 2021 y del Instituto Brasileño de Geografía y Estadística. Se realizó un análisis de las tendencias temporales de la tasa de incidencia mediante el software Joinpoint, calculando la variación porcentual anual media (AVP) y sus intervalos de confianza del 95% (IC95%). También se calculó la variación porcentual de las tasas de incidencia de 2020 a 2022 para comprobar si estaban por debajo o por encima de lo esperado para el año y el territorio.

**Resultados:**

De 2007 a 2022 se notificaron 13.890 nuevos casos de leishmaniasis visceral en niños y adolescentes del Nordeste de Brasil. Se identificaron cambios en las tendencias con la suma de los años de la pandemia de covid-19, de 2020 a 2022, en el Nordeste: VPAM (2007-2019) 0,44; IC95% -2,23; 3.18; VPAM (2007-2022) -7,95; IC95% -13,63; -1,88), Pernambuco: VPAM (2007-2019) 6,77; IC95% 2,04; 11,71; VPAM (2007-2022) -1,40; IC95% -8,75; 6,53 y Alagoas: VPAM (2007-2019) 11,51; IC95% 4,90; 18.53; VPAM (2007-2022) -1,33; IC95% -14,89; 14.40).

**Conclusión:**

El Nordeste y sus estados donde se observaron cambios en las tendencias temporales después de la pandemia de covid-19 son prioritarios para intensificar las acciones de combate y control de la enfermedad.

**Ethical aspects**

This research used public domain data and anonymized databases.

## Introduction

Leishmaniasis is a complex of diseases caused by intracellular protozoa of the genus Leishmania, which multiply inside macrophages, interfering in inflammatory modulation and overloading lymphatic organs essential for maintaining blood circulation (1). Leishmaniasis is divided into three diseases with distinct clinical manifestations: cutaneous, mucocutaneous and visceral (2). Visceral leishmaniasis is a neglected, zoonotic, rapidly progressive tropical disease caused in the Americas by the protozoan *Leishmania* (*L.) chagasi* (3), transmitted by Phlebotomine sandflies and with a higher incidence in developing countries (1). The clinical manifestations of the disease are systemic, and include fever, weight loss, lymphadenopathy, hyperpigmentation, hepatosplenomegaly and pancytopenia (3).

Considered endemic in 78 countries, visceral leishmaniasis is more prevalent in developing countries, mainly affecting economically disadvantaged populations (3) and is associated with difficulties in prevention, early detection and treatment by surveillance programs (4).

In 2016, Brazil was classified as a risk area for visceral leishmaniasis, and together with Peru and Colombia it accounted for approximately 15% of all cases in the world (5). Although visceral leishmaniasis is present in almost all regions of Brazil, the Northeast is the priority area for combating and controlling visceral leishmaniasis (6), accounting for 54.8% of cases of the disease in the country between 2001 and 2020 (7), and affecting especially children and adolescents (8-9).

Since the disease is endemic in Brazil, federal strategies are established to control visceral leishmaniasis, such as the distribution of insecticide collars for dogs to priority municipalities in 19 federative units. (10), which included the 9 states that make up the Northeast (11), as well as state-level action plans to intensify surveillance and control of the disease (12-13).

The incidence of visceral leishmaniasis in Brazil, especially in the Northeast region, has shown variations in recent decades (9), with a stationary trend in the region and increasing or decreasing trend in some federative units (14). These fluctuations appear to have been greater during the COVID-19 pandemic, declared in March 2020 by the World Health Organization. From 2020 to 2022, the COVID-19 pandemic caused a reduction in screenings, medical consultations and diagnoses (15-16), affecting especially neglected tropical diseases, both in Brazil (16-19) and abroad (20).

Despite the endemic scenario of visceral leishmaniasis in Brazil, especially in the Northeast, and the fact that children and adolescents are the most affected by the disease, there is a gap when it comes to the impact of the COVID-19 pandemic on the incidence of the disease in this age group.

Thus, this study aims to evaluate the impact of COVID-19 on the incidence of visceral leishmaniasis in children and adolescents living in the Northeast region of Brazil.

## Methods

### Study design

Time series study, based on population data on visceral leishmaniasis in children and adolescents aged less than one year to 19 years living in Northeast Brazil from 2007 to 2022. The Northeast region and its nine states were the units used for analysis in this study.

### Study Location

Brazil is a country located in South America with a population of 203,080,756 inhabitants. It is made up of five regions, including the Northeast region, which has a population of 54,658,515 inhabitants, according to the 2022 census conducted by the Brazilian Institute of Geography and Statistics (21).

### Data sources

Data on new cases of visceral leishmaniasis in individuals aged 0 to 19 years from 2007 to 2022 were collected from the Brazilian Information System for Notifiable Diseases, anonymized and made publicly available by the SUS (Brazilian Unified Health System) IT department of the Ministry of Health (22). This data were extracted from Individual Investigation Forms, filled out by healthcare professionals and subsequently entered into the Information Systems.

Demographic data were collected from the population residing in the Northeast region and its federative units from the 2010 (23) and 2022 (21) demographic censuses, made publicly available by the Brazilian Institute of Geography and Statistics, and from the study of population estimates by municipality, age and sex, from 2000 to 2021 (24). All data was collected on February 20, 2024.

### Study variables and indicators

Of the confirmed cases of visceral leishmaniasis in children and adolescents, only sociodemographic information was collected. Thus, the variables used were: year of notification and federative unit of residence. Age group filters, defined for children aged between 0 and 19 years, and entry type filters, defined for new cases only, were used.

From the sociodemographic data of the population residing in the Northeast region and its federative units, the following variables were derived: population residing in the Northeast region and population by Federative Unit. The data was filtered for the age group from 0 year to 19 years of age.

Three indicators were calculated for the analysis: annual incidence rate, average incidence rate and percentage change.

The annual incidence rate aims to identify the ratio between new cases in the population residing in the area in a year, and was calculated with the total number of new cases in the area in a year, divided by the population residing in the area in the same year and multiplying the quotient by 100 thousand.

The average incidence rate aims to verify the incidence rate of one area in a given period, being calculated as the arithmetic mean of the annual incidence rates of one specific area.

The percentage change aims to verify whether the incidence rate for a year is above or below what is expected for the same year, and has been used to assess the impact of COVID-19 on the incidence of diseases (14). It is calculated as follows:


$$\text{Variação percentual}=\frac{\text{Taxa de incidência observada}-\text{Taxa de incidência esperada}}{\text{Taxa de incidência esperada}}\times 100$$

Where the observed incidence rate is the annual incidence rate in the area and the expected incidence rate corresponds to the average of the incidence rates of the previous 5 years in that area.

### Statistical analysis

The analysis of temporal trends (25) was performed using the segmented linear regression method, with natural logarithmic transformation being performed, then the annual percentage variations were calculated and, after that, the average annual percentage variations (AAPV) and their 95% confidence intervals (95%CI). Increasing trends were considered those with p-value<0.050 and AAPV>0.000; decreasing trends were those with p-value<0.050 and APV<0.000; and stationary trends were those with p-value>0.050, regardless of the average annual percentage variation. To select the best models, Monte Carlo permutation analysis was used, with 999 permutations, with the use of the parametric method to calculate the p-value.

The analyses were performed using Joinpoint software v.5.0.2.

## Results

From 2007 to 2022, 13,890 new cases of visceral leishmaniasis were reported in children and adolescents in the Northeast region of Brazil, with an average incidence rate of 4.58 throughout the period and 2.13 when the last three years were observed. Regarding the frequencies of new cases, Maranhão (33.6%), Ceará (20%) and Bahia (18.5%) were the federative units with most of the disease notifications, while Maranhão (10.93), Piauí (7.68) and Ceará (5.70) had the highest average incidence rates throughout the period studied. In contrast, Paraíba (2%), Alagoas (2.9%) and Sergipe (3.0%) were responsible for the smallest numbers of notifications, while Alagoas (2.16), Pernambuco (1.87) and Paraíba (1.34) were the federative units with the lowest average incidence rates throughout the period studied (Table 1).

**Table 1:** Relative and absolute frequencies, and standard deviation of new cases of visceral leishmaniasis in children and adolescents, and average incidence rate in children and adolescents.Northeast region, 2007-2022

Federative Unit	n (%)	Mean (standard deviation)	Average incidence rate per 100,000	
			2007 to 2022	2020 to 2022
Northeast	13,890 (100.0%)	868.13 (293.58)	4.58	2.13
Alagoas	408 (2.9%)	25.50 (11.92)	2.16	2.04
Bahia	2,575 (18.5%)	160.94 (75.93)	3.34	1.44
Ceará	2,780 (20.0%)	173.75 (88.08)	5.79	1.95
Maranhão	4,674 (33.6%)	292.13 (115.80)	10.93	4.95
Paraíba	272 (2.0%)	17.00 (7.14)	1.34	0.89
Pernambuco	911 (6.6%)	56.94 (21.50)	1.87	1.43
Piauí	1,381 (9.9%)	86.31 (37.26)	7.68	3.12
Rio Grande do Norte	477 (3.4%)	29.81 (11.36)	2.75	1.26
Sergipe	412 (3.0%)	25.75 (9.17)	3.43	2.10

When the last 3 years were analyzed, during the COVID-19 pandemic Sergipe (2.10), Piauí (3.12) and Maranhão (4.95) were noted to be the federative units with the highest average incidence rates, while Rio Grande do Norte (1.26), Pernambuco (1.43) and Paraíba (0.89) had the lowest rates recorded in the period (Table 1).

When examining the incidence rates of each federative unit per year, it was noted that Maranhão was among the highest rates every year, occupying the position of highest incidence rate continuously since 2015 (Table 2).

**Table 2:** Annual incidence rates per of visceral leishmaniasis in children and adolescents. Northeast region, 2007-2022

Year	Territorial area									
	Alagoas	Bahia	Ceará	Maranhão	Paraíba	Pernambuco	Piauí	Rio Grande do Norte	Sergipe	Northeast
2007	1.45	2.51	9.05	8.20	1.00	1.55	12.32	2.65	5.37	4.72
2008	1.62	2.37	7.30	13:00	1.88	1.66	8.93	3.21	2.90	4.89
2009	1.79	4.59	10.52	10.52	0.88	1.30	8.59	3.77	3.05	5.53
2010	1.88	5.37	7.55	10.64	0.77	1.30	6.36	3.05	5.80	5.22
2011	2.22	4.53	8.13	12.03	1.73	1.67	9.02	3.95	3.23	5.54
2012	1.68	3.42	4.70	8.36	0.46	1.18	6.96	3.80	4.42	3.92
2013	1.30	4.09	7.28	17.35	1.17	1.19	9.89	2.93	3.67	5.88
2014	2.30	6.45	7.73	12.26	1.73	3.04	12.63	3.79	3.05	6.41
2015	2.25	4.32	6.88	11.57	1.75	2.71	8.91	1.87	3.07	5.26
2016	0.93	2.19	4.61	14.34	1.21	1.65	5.72	2.36	2.30	4.28
2017	2.42	3.61	4.44	17.46	1.96	2.80	9.75	3.92	3.70	5.77
2018	5.62	3.57	5.05	16.56	2.47	3.27	8.76	2.71	4.02	5.93
2019	3.04	2.16	3.59	7.72	1.83	2.36	5.61	2.25	4.06	3.52
2020	2.46	1.78	2.11	6.69	0.67	1.66	3.64	1.38	2.69	2.60
2021	2.31	1.58	1.42	4.04	0.94	1.39	2.00	1.30	1.43	1.88
2022	1.34	0.97	2.33	4.12	1.07	1.24	3.73	1.11	2.18	1.92

By comparing the average annual percentage variation of each territory from 2007 to 2019 with that from 2007 to 2022, it was possible to observe in which territories the trends were maintained and in which the trends changed when the incidence rates of the period of the COVID-19 pandemic, 2020, 2021 and 2022, were inserted. The Northeast showed a stationary trend from 2007 to 2019 (AAPV 0.44; 95%CI -2.23; 3.18; p-value 0.727) and a decreasing trend from 2007 to 2022 (AAPV -7.95; 95%CI -13.63; -1.88; p-value 0.011), as well as Sergipe, which presented a stationary trend in the period from 2007 to 2019 (AAPV -2.55; 95%CI -6.28; 1.32; p-value 0.173), but from 2007 to 2022 the identified trend was decreasing (AAPV -3.59; 95%CI -6.63; -0.44; p-value 0.028). Maranhão, Piauí, Rio Grande do Norte, Paraíba and Sergipe maintained their stationary trends, while Ceará was the only territory that remained with a decreasing trend. Pernambuco showed an increasing trend from 2007 to 2019 (AAPV 6.77; 95%CI 2.04; 11.71; p-value 0.009) and a stationary trend from 2007 to 2022 (AAPV -1.40; 95%CI -8.75; 6.53; p-value 0.720), and Alagoas showed an increasing trend (AAPV 11.51; 95%CI 4.90; 18.53; ppvalue 0.002) from 2007 to 2019, while in the period from 2007 to 2022 it presented a stationary trend (AAPV -1.33; 95%CI -14.89; 14.40; p-value 0.859) (Table 3).

**Table 3:** Temporal trends, average annual percentage variation (AAPV) and 95% confidence interval (95%CI) of the incidence of visceral leishmaniasis. Northeast region, 2007-2019 and 2007-2022

Variable	Period	AAPV (95%CI)	p-value
Northeast	2007-2019	0.44 (-2.23; 3.18)	0.727
	2007-2022	-7.95 (-13.63; -1.88)	0.011
Maranhão	2007-2019	3.46 (-0.63; 7.72)	0.090
	2007-2022	-8.28 (-17.31; 1.73)	0.102
Piauí	2007-2019	-1.69 (-5.45; 2.21)	0.355
	2007-2022	-3.02 (-6.59; 0.68)	0.101
Ceará	2007-2019	-5.76 (-8.86; -2.55)	0.002
	2007-2022	-6.68 (-9.52; -3.76)	<0.001
Rio Grande do Norte	2007-2019	-1.14 (-4.74; 2;59)	0.509
	2007-2022	-2.88 (-6.38; 0.75)	0.110
Paraíba	2007-2019	3.99 (-0.49; 8.67)	0.077
	2007-2022	1.48 (-3.03; 6.19)	0.500
Pernambuco	2007-2019	6.77 (2.04; 11.71)	0.009
	2007-2022	-1.40 (-8.75; 6.53)	0.720
Alagoas	2007-2019	11.51 (4.90; 18.53)	0.002
	2007-2022	-1.33 (-14.89; 14.40)	0.859
Sergipe	2007-2019	-2.55 (-6.28; 1.32)	0.173
	2007-2022	0.44 (0.12;1.56)	0.028
Bahia	2007-2019	-1.00 (-7.15; 5.57)	0.738
	2007-2022	-5.85 (-13.02; 1.91)	0.136

When the percentage variation the incidence of visceral leishmaniasis in children and adolescents in 2020, 2021 and 2022 were verified, it was identified that the incidence rate was below the expected value in the Northeast region (Percentage Variation in 2020=-49.62; Percentage Variation in 2021=-59.23; Percentage Variation in 2022 = -56.82) and in all Federative Units for all years (Table 4).

**Table 4:** Observed and expected incidence rates, and percentage variation in incidence of visceral leishmaniasis. Northeast region, 2020-2022

Territorial area	Observed incidence rate			Expected incidence rate			Percentage variation		
	2020	2021	2022	2020	2021	2022	2020	2021	2022
**Northeast region**	2.60	1.88	1.92	4.95	4.42	3.94	-49.62	-59.23	-56.82
Alagoas	2.46	2.31	1.34	2.85	2.89	3.17	-13.84	-20.05	-57.71
Bahia	1.78	1.58	0.97	3.17	2.66	2.54	-43.76	-40.86	-61.97
Ceará	2.11	1.42	2.33	4.92	3.96	3.32	-57.04	-64.11	-30.03
Maranhão	6.69	4.04	4.12	13.53	12.55	10.50	-50.55	-67.79	-60.77
Paraíba	0.67	0.94	1.07	1.84	1.63	1.57	-63.49	-42.54	-32.08
Pernambuco	1.66	1.39	1.24	2.56	2.35	2.30	-35.26	-40.61	-45.99
Piauí	3.64	2.00	3.73	7.75	6.70	5.95	-53.09	-70.13	-37.40
Rio Grande do Norte	1.38	1.30	1.11	2.62	2.52	2.31	-47.22	-48.67	-51.81
Sergipe	2.69	1.43	2.18	3.43	3.35	3.18	-21.69	-57.48	-31,41

## Discussion

The present study verified, for the first time, the impact of the COVID-19 pandemic on the incidence of visceral leishmaniasis in children and adolescents living in the Northeast region of Brazil.

The Northeast region of Brazil is one of the regions at high risk of visceral leishmaniasis (7) and is a priority for actions and studies on the disease. A 2023 article on the incidence of visceral leishmaniasis with the entire population diagnosed in the period from 2007 to 2020 (14) obtained findings similar to the analysis carried out in the present study in the period from 2007 to 2022, with a decreasing trend throughout the period.

A time series study from 2007 to 2020 identified increasing trends in Alagoas and stationary trends in Sergipe and Pernambuco (14), which in the present study showed a change in the direction of their trends when the years of the COVID-19 pandemic were added, with Alagoas and Pernambuco showing a stationary trend, while Sergipe showed a decreasing trend.

Furthermore, social determinants of health, such as factors related to basic sanitation, housing, socioeconomic, cultural, ethnic-racial status, among others, are related to the incidence of this disease in more vulnerable populations, such as in the Brazilian Northeast. Nutritional deficit and immunological immaturity may explain the high incidence of the disease among children in some regions of Brazil, making the relevance of the study of visceral leishmaniasis in this age group evident (26).

As seen in studies that used temporal trend analysis techniques to verify the impact of COVID-19 on various diseases (16,19), the incidence rates of some federative units showed decreasing or stationary trends in the last years of the study.

When comparing the periods from 2007 to 2019 and from 2007 to 2022, the changes identified in the trends can be attributed to changes in the functioning of the Unified Health System (SUS) during the COVID-19 pandemic, due to the reduction in diagnoses, medical consultations and screening, especially in regions with greater social vulnerability (15), since the reduction of these processes can cause a decrease in the number of cases diagnosed in children and adolescents, and thus impact the reduction of the incidence rates of visceral leishmaniasis in this population.

The reorganization of the public health system due to social distancing measures limited users› access to health services. This, associated with the suspension of health promotion activities, the loss of medication distribution and patients› fear of going to health units, potential transmission points for COVID-19, had a negative impact on the surveillance of neglected tropical diseases (27), such as visceral leishmaniasis.

This change in the operations of the SUS (Brazilian Unified Health System) (15) was also in line with what was observed in the percentage variation in the incidence rates of visceral leishmaniasis in children and adolescents, since there was a reduction in the number of cases diagnosed in all federative units of the Northeast region, with incidence rates remaining below what was expected for the period of the COVID-19 pandemic.

This study had limitations due to the ecological fallacy, since it is a population-based study, making it impossible to make inferences at the individual level; the quality of disease notification data, due to underreporting and blank/ignored data; and the use of aggregated indicators, which does not allow the identification of internal factors of the areas that are related to the object of the study.

Thus, the impact of the COVID-19 pandemic on the incidence of visceral leishmaniasis in children and adolescents living in the Northeast of Brazil was analyzed, enabling the identification of important information, namely: 1) the COVID-19 pandemic impacted the incidence of visceral leishmaniasis in the Northeast, Alagoas and Sergipe, causing changes in the direction of trends; 2) Maranhão is an important area for the control of visceral leishmaniasis in children and adolescents in the region, since it has led the incidence rates in the last 8 years; 3) despite the drop in incidence rates, those federative units that have a stationary trend or that have shown changes in the direction of their trends need to focus on epidemiological surveillance and improve programs to combat and control the disease in children and adolescents. Thus, this study makes an unprecedented contribution to research on visceral leishmaniasis in Brazil, and the impact of the COVID-19 pandemic in terms of incidence and of the most vulnerable age groups.

## Data Availability

The databases used in the research are available at the following link: https://osf.io/2jbyg/ (28).
