# Supplementary material for: Impact of COVID-19 on the incidence of visceral leishmaniasis in children and adolescents in the Northeast region, 2007-2022: time series study
Source: Epidemiol Serv Saude. 2025 Jun 13;34:e20240382. doi: 10.1590/S2237-96222025v34e20240382.en (PMC12176430; doi:10.1590/S2237-96222025v34e20240382.en)
Supplement: Translation

# Translation: Impacto da covid-19 na incidência de leishmaniose visceral em crianças e adolescentes na região Nordeste, 2007-2022: estudo de séries temporais

**DOI:** 10.1590/S2237-96222025v34e20240382.pt

**Authors:** Pedro Henrique Ferreira dos Santos, Bruna Rykelly Ramos dos Santos, Karol Fireman de Farias

**Affiliations:** 1 Universidade Federal de Alagoas, Arapiraca, AL, Brasil

**Keywords:** Leishmaniose Visceral, Saúde da Criança, Epidemiologia, Doenças Negligenciadas, Saúde Pública

## Abstract

**Objetivo:**

Avaliar o impacto da covid-19 na incidência de leishmaniose visceral em crianças e adolescentes residentes na região Nordeste do Brasil.

**Métodos:**

Estudo de séries temporais com dados do Sistema de Informação de Agravos de Notificação, do estudo de estimativas populacionais por município, idade e sexo de 2000 a 2021, e do Instituto Brasileiro de Geografia e Estatística. Foi realizada análise das tendências temporais da taxa de incidência utilizando o software Joinpoint, calculando-se a variação percentual anual média (VPAM) e seus intervalos de confiança de 95% (IC95%). Também foi calculada a variação percentual das taxas de incidência dos anos de 2020 a 2022, para verificar se ficaram abaixo ou acima do esperado para o ano e território.

**Resultados:**

De 2007 a 2022 foram notificados 13.890 novos casos de leishmaniose visceral em crianças e adolescentes no Nordeste do Brasil. Foram identificadas alterações nas tendências com a adição dos anos da pandemia de covid-19, de 2020 a 2022, no Nordeste: VPAM (2007-2019) 0,44; IC95% -2,23; 3,18; VPAM (2007-2022) -7,95; IC95% -13,63; -1,88), Pernambuco: VPAM (2007-2019) 6,77; IC95% 2,04; 11,71; VPAM (2007-2022) -1,40; IC95% -8,75; 6,53 e Alagoas: VPAM (2007-2019) 11,51; IC95% 4,90; 18,53; VPAM (2007-2022) -1,33; IC95% -14,89; 14,40).

**Conclusão:**

O Nordeste e seus estados onde foram observadas alterações nas tendências temporais após a pandemia de covid-19 são prioritários para a intensificação das ações de combate e controle da doença.

**Aspectos éticos**

Esta pesquisa utilizou bancos de dados de domínio público e anonimizados.

## Introdução

A leishmaniose é um complexo de doenças causadas por protozoários intracelulares do gênero Leishmania, que se multiplicam no interior de macrófagos, interferindo na modulação inflamatória e sobrecarregando órgãos linfáticos essenciais para a manutenção da circulação sanguínea (1). A leishmaniose divide-se em três doenças com manifestações clínicas distintas: cutânea, mucocutânea e visceral (2). A leishmaniose visceral é uma doença tropical negligenciada, zoonótica, de progressão rápida, causada nas Américas pelo protozoário *Leishmania* (*L.) chagasi* (3), transmitida por Flebotomíneos e com maior incidência em países em desenvolvimento (1). As manifestações clínicas da doença são sistêmicas, e incluem febre, perda de peso, linfadenopatia, hiperpigmentação, hepatoesplenomegalia e pancitopenia (3).

Considerada endêmica em 78 países, a leishmaniose visceral incide mais naqueles em desenvolvimento, atingindo principalmente as populações economicamente desfavorecidas (3) e estando associada à problemas em programas de vigilância para prevenção, detecção precoce e tratamento (4).

Em 2016, o Brasil foi classificado como áreas de risco de leishmaniose visceral, e juntamente com Peru e Colômbia representou cerca de 15% de todos os casos do mundo (5). Embora a leishmaniose visceral esteja presente em quase todas as regiões do Brasil, é o Nordeste que representa a área prioritária para o combate e controle da leishmaniose visceral (6), tendo concentrado 54,8% dos casos da doença no país entre os anos 2001 e 2020 (7), e acometendo especialmente as crianças e adolescentes (8-9).

Uma vez que a doença é de caráter endêmico no Brasil, estratégias à nível federal são estabelecidas para controle da leishmaniose visceral, como a distribuição de coleiras com inseticida para cachorros para municípios prioritários em 19 unidades federativas (10), onde se incluíram os 9 estados que compõem o Nordeste (11), bem como planos de ação à nível estadual para intensificar a vigilância e o controle da doença (12-13).

O cenário da incidência de leishmaniose visceral no Brasil, especialmente na região Nordeste, mostra variações nas últimas décadas (9), com tendência estacionária na região, crescentes ou decrescentes em algumas unidades federativas (14). Estas flutuações parecem terem sido maiores durante a pandemia de covid-19, decretada em março de 2020 pela Organização Mundial da Saúde. A pandemia de covid-19 no período de 2020 a 2022, causou redução das triagens, consultas médicas e diagnósticos (15-16), afetando especialmente as doenças tropicais negligenciadas, tanto no Brasil (16-19), quanto no exterior (20).

Apesar do cenário endêmico da leishmaniose visceral no Brasil, principalmente no Nordeste, e do fato de crianças e adolescentes serem os mais afetados pela doença, há uma lacuna quando se trata do impacto da pandemia de covid-19 na incidência da doença neste grupo.

Desta forma, este estudo tem como objetivo avaliar o impacto da covid-19 na incidência de leishmaniose visceral em crianças e adolescentes residentes na região Nordeste do Brasil.

## Métodos

### Delineamento

Estudo de séries temporais, baseado em dados populacionais de leishmaniose visceral em crianças e adolescentes de menos de um ano a 19 anos residentes no Nordeste do Brasil do período de 2007 a 2022. Teve como unidades de análise a região Nordeste e seus nove estados.

### Local do Estudo

O Brasil, é um país localizado na América do Sul e conta com 203.080.756 habitantes, composto por cinco regiões, dentre elas, a região Nordeste, que é contabilizou 54.658.515, segundo o censo realizado pelo Instituto Brasileiro de Geografia e Estatística habitantes no censo de 2022 (21).

### Fontes de Dados

Coletaram-se os dados sobre os casos novos de leishmaniose visceral em indivíduos com faixa etária de 0 a 19 anos no período de 2007 a 2022 do Sistema de Informação sobre Agravos de Notificação, anonimizados e disponibilizados publicamente pelo departamento de informática do SUS, do Ministério da Saúde (22). Esses dados são provenientes de Fichas Individuais de Investigação, preenchidas por profissionais de saúde e posteriormente digitadas nos Sistemas de Informação.

Coletaram-se os dados demográficos da população residente na região Nordeste e de suas unidades federativas dos censos demográficos de 2010 (23) e 2022 (21), disponibilizados publicamente pelo Instituto Brasileiro de Geografia e Estatística, e do estudo de estimativas populacionais por município, idade e sexo de 2000 a 2021 (24). Todos os dados foram coletados no dia 20 de fevereiro de 2024.

### Variáveis do estudo e indicadores

Dos casos confirmados de leishmaniose visceral em crianças e adolescentes, foram coletados apenas as informações sociodemográficas. Desta forma as variáveis utilizadas foram: ano de notificação e unidade federativa de residência. Sendo utilizados os filtros de faixa etária, definido para casos de menores de 1 ano a 19 anos, e de tipo de entrada, sendo definida apenas para casos novos.

Dos dados sociodemográficos da população residente na região Nordeste e de suas unidades federativas, foram utilizadas as seguintes variáveis: população residente na região Nordeste e população residente por Unidade Federativa. Os dados foram filtrados para a faixa etária de menores de 1 ano a 19 anos.

Três indicadores foram calculados para a análise: a taxa de incidência anual, taxa de incidência média e variação percentual.

A taxa de incidência anual tem como objetivo identificar a razão entre novos casos na população residente nos territórios em um ano, e foi calculada com o total de novos casos no território num ano, dividido pela população residente no território no mesmo ano e multiplicando o quociente por 100 mil.

A taxa de incidência média tem como objetivo verificar a taxa de incidência de um território em um determinado período, sendo calculada como a média aritmética das taxas de incidência anuais de um território.

A variação percentual tem como objetivo verificar se a taxa de incidência de um ano está acima ou abaixo do esperado para o mesmo ano, e vem sendo utilizada para avaliar o impacto da covid-19 na incidência de doenças (14), sendo calculada da seguinte forma:


$$\text{Variação percentual}=\frac{\text{Taxa de incidência observada}-\text{Taxa de incidência esperada}}{\text{Taxa de incidência esperada}}\times 100$$

Onde a taxa de incidência observada é a taxa de incidência anual no território e a taxa de incidência esperada corresponde à média das taxas de incidência dos 5 anos anteriores no território.

### Análise estatística

A análise de tendências temporais (25) foi realizada através do método de regressão linear segmentada, sendo realizada transformação logarítmica natural, em seguida foram calculadas as variações percentuais anuais e, após isso, as variações percentuais anuais médias (VPAM) e seus intervalos de confiança de 95% (IC95%). Foram consideras tendências crescentes aquelas com p-valor<0,050 e VPAM>0,000, decrescentes aquelas com p-valor<0,050 e VPA<0,000, e estacionárias aquelas com p-valor>0,050, independente da variação percentual anual média. Para a seleção dos melhores modelos, foi utilizada a análise de permutação de Monte Carlo, com 999 permutações, utilizando o método paramétrico para o cálculo do p-valor.

As análises foram realizadas utilizando os softwares Joinpoint v.5.0.2.

## Resultados

De 2007 a 2022 foram notificados 13.890 novos casos de leishmaniose visceral em crianças e adolescentes na região Nordeste do Brasil, com taxas de incidência média de 4,58 durante todo o período e de 2,13 quando observados os três últimos anos. Observando-se as frequências dos casos novos, o Maranhão (33,6%), o Ceará (20%) e a Bahia (18,5%) foram as unidades federativas que mais tiveram notificações da doença, já o Maranhão (10,93), o Piauí (7,68) e o Ceará (5,70) tiveram as maiores taxas de incidência média durante todo o período estudado. Em contrapartida, Paraíba (2%), Alagoas (2,9%) e Sergipe (3,0%) foram responsáveis pelas menores parcelas das notificações, enquanto Alagoas (2,16), Pernambuco (1,87) e Paraíba (1,34) foram as unidades federativas com as menores taxas de incidência média em todo o período estudado (Tabela 1).

**Tabela 1:** Frequências relativa e absoluta, e desvio padrão dos casos novos de leishmaniose visceral em crianças e adolescentes, e taxa de incidência média em crianças e adolescentes. Nordeste, 2007-2022

Unidade Federativa	n (%)	Média (desvio padrão)	Taxa de incidência média por 100 mil	
			2007 a 2022	2020 a 2022
Nordeste	13.890 (100,0%)	868,13 (293,58)	4,58	2,13
Alagoas	408 (2,9%)	25,50 (11,92)	2,16	2,04
Bahia	2.575 (18,5%)	160,94 (75,93)	3,34	1,44
Ceará	2.780 (20,0%)	173,75 (88,08)	5,79	1,95
Maranhão	4.674 (33,6%)	292,13 (115,80)	10,93	4,95
Paraíba	272 (2,0%)	17,00 (7,14)	1,34	0,89
Pernambuco	911 (6,6%)	56,94 (21,50)	1,87	1,43
Piauí	1.381 (9,9%)	86,31 (37,26)	7,68	3,12
Rio Grande do Norte	477 (3,4%)	29,81 (11,36)	2,75	1,26
Sergipe	412 (3,0%)	25,75 (9,17)	3,43	2,10

Examinando os últimos 3 anos, durante a pandemia de covid-19, foi identificado que Sergipe (2,10), Piauí (3,12) e Maranhão (4,95) foram as unidades federativas com as maiores taxas de incidência média, enquanto Rio Grande do Norte (1,26), Pernambuco (1,43) e Paraíba (0,89) tiveram as menores taxas registradas no período (Tabela 1).

Quando examinadas as taxas de incidência de cada unidade federativa por ano, percebeu-se que o Maranhão figurou entre as maiores taxas todos os anos, ocupando o posto de maior taxa de incidência de maneira contínua desde 2015 (Tabela 2).

**Tabela 2:** Taxas de incidência anuais de leishmaniose visceral em crianças e adolescentes. Nordeste, 2007-2022

Ano	Território									
	Alagoas	Bahia	Ceará	Maranhão	Paraíba	Pernambuco	Piauí	Rio Grande do Norte	Sergipe	Nordeste
2007	1,45	2,51	9,05	8,20	1,00	1,55	12,32	2,65	5,37	4,72
2008	1,62	2,37	7,30	13,00	1,88	1,66	8,93	3,21	2,90	4,89
2009	1,79	4,59	10,52	10,52	0,88	1,30	8,59	3,77	3,05	5,53
2010	1,88	5,37	7,55	10,64	0,77	1,30	6,36	3,05	5,80	5,22
2011	2,22	4,53	8,13	12,03	1,73	1,67	9,02	3,95	3,23	5,54
2012	1,68	3,42	4,70	8,36	0,46	1,18	6,96	3,80	4,42	3,92
2013	1,30	4,09	7,28	17,35	1,17	1,19	9,89	2,93	3,67	5,88
2014	2,30	6,45	7,73	12,26	1,73	3,04	12,63	3,79	3,05	6,41
2015	2,25	4,32	6,88	11,57	1,75	2,71	8,91	1,87	3,07	5,26
2016	0,93	2,19	4,61	14,34	1,21	1,65	5,72	2,36	2,30	4,28
2017	2,42	3,61	4,44	17,46	1,96	2,80	9,75	3,92	3,70	5,77
2018	5,62	3,57	5,05	16,56	2,47	3,27	8,76	2,71	4,02	5,93
2019	3,04	2,16	3,59	7,72	1,83	2,36	5,61	2,25	4,06	3,52
2020	2,46	1,78	2,11	6,69	0,67	1,66	3,64	1,38	2,69	2,60
2021	2,31	1,58	1,42	4,04	0,94	1,39	2,00	1,30	1,43	1,88
2022	1,34	0,97	2,33	4,12	1,07	1,24	3,73	1,11	2,18	1,92

Ao se comparar a variação percentual anual média de cada território do período de 2007 a 2019 com a do período de 2007 a 2022, foi possível observar em quais territórios as tendências foram mantidas e em quais as tendências foram alteradas quando inseridas as taxas de incidência do período da pandemia de covid-19, 2020, 2021 e 2022. O Nordeste apresentou tendência estacionária de 2007 a 2019 (VPAM 0,44; IC95% -2,23; 3,18; p-valor 0,727) e decrescente de 2007 a 2022 (VPAM -7,95; IC95% -13,63; -1,88; p-valor 0,011), bem como Sergipe, que apresentou tendência estacionária no período de 2007 a 2019 (VPAM -2,55; IC95% -6,28; 1,32; p-valor 0,173), mas de 2007 a 2022 a tendência identificada foi decrescente (VPAM -3,59; IC95% -6,63; -0,44; p-valor 0,028). Maranhão, Piauí, Rio Grande do Norte, Paraíba e Sergipe mantiveram suas tendências estacionárias, enquanto o Ceará foi o único território que permaneceu com tendência decrescente. Já Pernambuco apresentou tendência crescente de 2007 a 2019 (VPAM 6,77; IC95% 2,04; 11,71; p-valor 0,009) e estacionária de 2007 a 2022 (VPAM -1,40; IC95% -8,75; 6,53; p-valor 0,720), e Alagoas de 2007 a 2019 apresentou tendência crescente (VPAM 11,51; IC95% 4,90; 18,53; p-valor 0,002), enquanto no período de 2007 a 2022 apresentou tendência estacionária (VPAM -1,33; IC95% -14,89; 14,40; p-valor 0,859) (Tabela 3).

**Tabela 3:** Tendências temporais, variação percentual anual média (VPAM) e intervalo de confiança de 95% (IC95%), da incidência de leishmaniose visceral. Nordeste, 2007-2019 e 2007-2022

Variável	Período	VPAM (IC95%)	p-valor
Nordeste	2007-2019	0,44 (-2,23; 3,18)	0,727
	2007-2022	-7,95 (-13,63; -1,88)	0,011
Maranhão	2007-2019	3,46 (-0,63; 7,72)	0,090
	2007-2022	-8,28 (-17,31; 1,73)	0,102
Piauí	2007-2019	-1.69 (-5,45; 2,21)	0,355
	2007-2022	-3,02 (-6,59; 0,68)	0,101
Ceará	2007-2019	-5,76 (-8,86; -2,55)	0,002
	2007-2022	-6,68 (-9.52; -3,76)	<0,001
Rio Grande do Norte	2007-2019	-1,14 (-4,74; 2,59)	0,509
	2007-2022	-2,88 (-6,38; 0,75)	0,110
Paraíba	2007-2019	3,99 (-0,49; 8,67)	0,077
	2007-2022	1,48 (-3,03; 6,19)	0,500
Pernambuco	2007-2019	6,77 (2,04; 11,71)	0,009
	2007-2022	-1,40 (-8,75; 6,53)	0,720
Alagoas	2007-2019	11,51 (4,90; 18,53)	0,002
	2007-2022	-1,33 (-14,89; 14,40)	0,859
Sergipe	2007-2019	-2,55 (-6,28; 1,32)	0,173
	2007-2022	-3,59 (-6,63; -0,44)	0,028
Bahia	2007-2019	-1,00 (-7,15; 5,57)	0,738
	2007-2022	-5,85 (-13,02; 1,91)	0,136

Quando verificadas as variações percentuais da incidência de leishmaniose visceral em crianças e adolescentes nos anos 2020, 2021 e 2022, identificou-se que a taxa de incidência ficou abaixo do valor esperado na região Nordeste (Variação Percentual de 2020=-49,62; Variação Percentual de 2021=-59,23; Variação Percentual de 2022=-56,82) e em todas as unidades federativas em todos os anos (Tabela 4).

**Tabela 4:** Taxas de incidência observadas e esperadas, e variação percentual de incidência de leishmaniose visceral. Nordeste, 2020-2022

Território	Taxa de incidência observada			Taxa de incidência esperada			Variação percentual		
	2020	2021	2022	2020	2021	2022	2020	2021	2022
Nordeste	2,60	1,88	1,92	4,95	4,42	3,94	-49,62	-59,23	-56,82
Alagoas	2,46	2,31	1,34	2,85	2,89	3,17	-13,84	-20,05	-57,71
Bahia	1,78	1,58	0,97	3,17	2,66	2,54	-43,76	-40,86	-61,97
Ceará	2,11	1,42	2,33	4,92	3,96	3,32	-57,04	-64,11	-30,03
Maranhão	6,69	4,04	4,12	13,53	12,55	10,50	-50,55	-67,79	-60,77
Paraíba	0,67	0,94	1,07	1,84	1,63	1,57	-63,49	-42,54	-32,08
Pernambuco	1,66	1,39	1,24	2,56	2,35	2,30	-35,26	-40,61	-45,99
Piauí	3,64	2,00	3,73	7,75	6,70	5,95	-53,09	-70,13	-37,40
Rio Grande do Norte	1,38	1,30	1,11	2,62	2,52	2,31	-47,22	-48,67	-51,81
Sergipe	2,69	1,43	2,18	3,43	3,35	3,18	-21,69	-57,48	-31,41

## Discussão

O presente estudo verificou, pela primeira vez, o impacto da pandemia de covid-19 na incidência de leishmaniose visceral em crianças e adolescentes residentes na região Nordeste do Brasil.

A região Nordeste do Brasil é uma das regiões de alto risco de leishmaniose visceral (7) e prioritária para ações e estudos sobre a doença. Um artigo de 2023 sobre a incidência de leishmaniose visceral com toda a população diagnosticada no período de 2007 a 2020 (14) obteve achados semelhantes à análise realizada no presente estudo no período de 2007 a 2022, com tendência decrescente em todo o período.

Um estudo de séries temporais do período de 2007 a 2020 identificou tendências crescentes em Alagoas e estacionária em Sergipe e Pernambuco (14), que no presente estudo apresentaram mudança no sentido de suas tendências quando adicionados os anos da pandemia de covid-19, com Alagoas e Pernambuco apresentando tendência estacionária, enquanto Sergipe apresentou tendência decrescente.

Ademais, os determinantes sociais da saúde, como fatores relacionados ao saneamento básico, moradia, situação socioeconômica, cultural, étnico-racial, entre outros, estão relacionados com a incidência dessa doença em populações mais vulneráveis, como no Nordeste brasileiro. Déficit nutricional e imaturidade imunológica pode explicar a alta incidência da doença entre as crianças em algumas regiões do Brasil, ficando evidente a relevância do estudo da leishmaniose visceral nesta faixa etária (26).

Assim como visto em estudos que utilizaram técnicas de análise de tendências temporais para verificar o impacto da covid-19 em diversas doenças (16,19), as taxas de incidências de algumas unidades federativas apresentaram tendências decrescentes ou estacionárias nos últimos anos do estudo.

Estas mudanças identificadas nas tendências quando comparados os períodos de 2007 a 2019 e de 2007 a 2022, podem ser atribuídas a alterações no funcionamento do Sistema Único de Saúde (SUS) durante a pandemia de covid-19, devido à redução de diagnósticos, consultas médicas e triagem, principalmente em regiões com maior vulnerabilidade social (15), uma vez que a redução desses processos pode causar diminuição na quantidade de casos diagnosticados em crianças e adolescentes, e assim impactar na redução das taxas de incidência de leishmaniose visceral nessa população.

A reorganização do sistema de saúde devido às medidas de distanciamento social, limitou a busca dos usuários aos serviços de saúde. Isto associado a suspensão de atividades de promoção à saúde, ao prejuízo nas distribuições de medicamentos e ao medo dos pacientes de irem às unidades de saúde, possíveis pontos de transmissão do covid-19, impactou negativamente na vigilância doenças tropicais negligenciadas (27), como a leishmaniose visceral.

Essa alteração no funcionamento do SUS (15) também foi ao encontro do observado na variação percentual das taxas de incidência de leishmaniose visceral em crianças e adolescentes, uma vez que houve redução no número de casos diagnosticados em todas as unidades federativas da região Nordeste, com as taxas de incidência ficando abaixo do esperado para o período da pandemia de covid-19.

Este estudo teve como limitações a falácia ecológica, uma vez que é um estudo de base populacional, impossibilitando inferências ao nível de indivíduos; a qualidade dos dados das notificações da doença, devido à subnotificação e dados em branco/ignorados; e a utilização de indicadores agregados, o que não permite a identificação de fatores internos dos territórios que se relacionam com o objeto do estudo.

Desta forma, o impacto da pandemia de covid-19 na incidência de leishmaniose visceral em crianças e adolescentes residentes no Nordeste do Brasil foi verificado, possibilitando a identificação de informações importantes, sendo elas: 1) a pandemia de covid-19 impactou a incidência de leishmaniose visceral no Nordeste, Alagoas e Sergipe, causando alterações no sentido das tendências; 2) o Maranhão é uma área importante para o controle da leishmaniose visceral em crianças e adolescentes na região, uma vez que lidera as taxas de incidência nos últimos 8 anos; 3) apesar da queda nas taxas de incidência, aquelas unidades federativas que possuem tendência estacionária ou que apresentaram alterações nos sentidos de suas tendências necessitam enfoque na vigilância epidemiológica e melhoria nos programas de combate e controle da doença em crianças e adolescentes. Desta forma, este estudo contribui de maneira inédita para as pesquisas sobre leishmaniose visceral no Brasil, e o impacto da pandemia de covid-19 quanto a incidência e grupos etários mais vulneráveis.

## Data Availability

Os bancos de dados utilizados na pesquisa estão disponíveis no seguinte link: https://osf.io/2jbyg/ (28).
